# Lowered Serum Testosterone Concentration Is Associated With Enhanced Inflammation and Worsened Lipid Profile in Men

**DOI:** 10.3389/fendo.2021.735638

**Published:** 2021-09-09

**Authors:** Marcin Grandys, Joanna Majerczak, Justyna Zapart-Bukowska, Krzysztof Duda, Jan K. Kulpa, Jerzy A. Zoladz

**Affiliations:** ^1^Department of Muscle Physiology, Institute of Basic Sciences, Faculty of Rehabilitation, University School of Physical Education, Krakow, Poland; ^2^Faculty of Health Sciences, Jagiellonian University Medical College, Krakow, Poland; ^3^Department of Neurobiology, Poznan University of Physical Education, Poznań, Poland; ^4^Department of Nursing, Institute of Health Protection, The State Higher School of Vocational Education, Tarnow, Poland; ^5^Department of Clinical Biochemistry, Centre of Oncology, Maria Sklodowska-Curie Memorial Institute, Cracow Branch, Krakow, Poland

**Keywords:** androgens, inflammaging, adiposity, exercise training, alpha-1-acid glycoprotein

## Abstract

The negative relationship between testosterone and inflammatory cytokines has been reported for decades, although the exact mechanisms of their interactions are still not clear. At the same time, little is known about the relation between androgens and acute phase proteins. Therefore, in this investigation, we aimed to study the relationship between androgen status and inflammatory acute phase reactants in a group of men using multi-linear regression analysis. Venous blood samples were taken from 149 men ranging in age from 18 to 77 years. Gonadal androgens [testosterone (T) and free testosterone (fT)], acute phase reactants [C-reactive protein (CRP), ferritin (FER), alpha-1-acid glycoprotein (AAG), and interleukin-6 (IL-6)], cortisol (C), and lipid profile concentrations were determined. It was demonstrated that the markers of T and fT were negatively correlated with all acute phase proteins (CRP, FER, and AAG; *p* < 0.02) and the blood lipid profile [total cholesterol (TC), low-density lipoprotein (LDL), and triglycerides (TG); *p* < 0.03]. Multivariate analysis showed that T, fT, and the fT/C ratio were inversely correlated with the CRP, AAG, and FER concentrations independently of age and blood lipids. When adjustment for BMI was made, T, fT, and the fT/C ratio were negatively correlated with the AAG concentrations only. In addition, it was demonstrated that gonadal androgens were positively correlated with physical activity level (*p* < 0.01). We have concluded that a lowered serum T concentration may promote inflammatory processes independently of adipose tissue and age through a reduced inhibition of inflammatory cytokine synthesis, which leads to enhanced acute phase protein production. Therefore, a low serum T concentration appears to be an independent risk factor in the development of atherosclerosis and cardiovascular diseases. Moreover, the positive correlation between testosterone and physical activity level suggests that exercise training attenuates the age-related decrease in gonadal androgens and, in this way, may reduce the enhancement of systemic low-grade inflammation in aging men.

## Introduction

Age-related upregulation of the inflammatory response (described as “inflamm-aging”) (1) and the worsening of the blood lipid profile are of great importance because these changes are linked to atherosclerosis, enhanced cardiovascular risk, and the development of metabolic syndrome. In the recent comprehensive review on the inflammatory etiology of cardiovascular diseases by Ruscica et al. (2), the role of evaluation of the pro- and anti-inflammatory profiles for appropriate guidelines and treatment of this disease was pointed out. Simultaneously, it should be mentioned that an enhanced pro-inflammatory status after the fifth to the sixth decade of life (1) is accompanied by a decrease in testosterone (T) concentration, and some have suggested that changes in the inflammatory markers and testosterone in aging men are causally linked (3).

In recent years, connections between testosterone and the inflammatory process have been widely studied [for a review, see (4)], although the existence of the bidirectional mechanisms between the immune and endocrine systems was reported at least 20 years ago (5). This concept has a strong scientific foundation since androgens have been shown to regulate the inflammatory response (6) by suppressing pro-inflammatory leukotriene biosynthesis (7), decreasing pro-inflammatory mediators, and increasing anti-inflammatory cytokines, leading to a state of reduced inflammation (8). Moreover, the claims on the anti-inflammatory effects of T are based on observations of the enhanced inflammatory cytokine levels in hypogonadal men and the reduced inflammatory markers in T supplementation studies (4). On the other hand, an inflammatory process as a manifestation of increased oxidative stress may negatively influence the androgen level (9), both through direct disruption of the reproductive tissue and through the detrimental effect on the regulatory mechanisms of the hypothalamic–pituitary–gonadal (HPG) axis.

The interactions between androgens and inflammation may be influenced by adipose tissue because it is well known that the inflammatory process results from an imbalance between the pro- and antioxidant systems often related to dysfunctional adipose tissue (10). We have recently demonstrated that the BMI and body fat percentage correlated positively with the inflammatory and oxidative stress markers in men (11), which could explain the age-related increase in inflammation and oxidative stress that led to a decline in endothelial function and an increase in arterial stiffness. In addition, Bobjer et al. (12) reported that a low T concentration was associated with elevated tumor necrosis factor alpha (TNF-α) and pro-inflammatory chemokines in relatively young men without any metabolic disorders and disease. It was also demonstrated that obesity may result in hypogonadism and T supplementation interventions, leading to a eugonadal state, appear to decrease the body fat content (13).

The possible influence of body fat content on the relationship between androgen and inflammatory status also indicates that the level of physical activity could be of paramount importance in this connection. There is a fair amount of data showing that regular exercise training may attenuate inflammation (14, 15), even in serious neurodegenerative diseases (16). Some authors postulated that this effect may be attributable to the training-induced reduction in adipose tissue content (14), however, it was also suggested that exercise may lead to anti-inflammatory effects that are independent of weight loss (17). Although the mechanisms of this important outcome of exercise training in aging are at best unclear, one possible explanation lies in the fact that both gonadal androgens and inflammatory status may be affected by the applied training program. What is interesting is that a heavy exercise training program is thought to unfavorably affect the T concentration (18) and the pro- and anti-inflammatory balance (19), whereas moderate training loads were linked to both enhanced gonadal androgen concentrations (20) and anti-inflammatory state (21).

Studies concerning the relationship between androgens and inflammation have so far mainly focused on the inflammatory cytokines such as interleukin-1 (IL-1), IL-6, and TNF-α (4). Far less is known about the possible interactions between testosterone and acute phase proteins [C-reactive protein (CRP), ferritin (FER), and alpha-1-acid glycoprotein (AAG)], which are all important for inflammatory responses and are frequently assayed in standard laboratory tests. For this reason, we aimed to study the relationship between androgen status and inflammatory acute phase reactants (CRP, FER, and AAG) in a moderately large group of men using multi-linear regression analysis. We wanted to verify the hypothesis that a higher androgen status is related to a better inflammatory profile independently of confounders such as age, BMI, and lipid profile. Because of the aforementioned potential effect of exercise on this relationship, we were also interested in determining the importance of the level of physical activity of the studied subjects on their androgen profile.

## Material and Methods

### Subjects

A group of 149 men ranging in age from 18 to 77 years were investigated. The experimental data presented in this manuscript were gathered from our previous research projects (11, 20, 22–24), but the majority of the subjects (*n* = 94) involved in this investigation participated in the study by Majerczak et al. (11). Nevertheless, the primary goals of this work and the main analyses reported herein are novel and do not overlap those presented in prior publications. The inclusion criteria for the men selected in this study were as follows: minimum 18 and maximum 80 years old, absence of serious medical illness, no drug or alcohol abuse in the past, and no smoking in the past 10 years. The participants underwent a standard medical evaluation and a routine blood examination, and if these tests showed no contraindications, they were included in the study. Each participant responded to lifestyle questionnaires including occupational, dietary, and physical activity and training characteristics. The group consisted of both sedentary and trained men, and the mean actual level of physical activity amounted to 5.1 ± 4.8 h per week. Some of the trained men were recreationally active and some were professionally trained in both sprint and endurance events, currently or in the past (former athletes). The basic anthropometric, hematological, and blood biochemical characteristics of the studied group are given in **Table 1**. Ethical approval for the experimental procedures was obtained from the Local Ethical Committee at the Regional Medical Chamber in Krakow, Poland (opinion no. 48/KBL/OIL/2009), and the study protocol was conducted in accordance with the Declaration of Helsinki. All volunteers were fully informed about the aim of this study and gave written consent to take part in the investigation.

**Table 1:** Basic anthropometric, hematological, biochemical, and hormonal parameters of the studied subjects (*n* = 149).

	$\bar{x}$ ± SD	95% CI	Min–Max
*Anthropometric data*			
Age (years)	38.6 ± 19.1	35.5–41.6	18.0–77.0
Height (cm)	178.0 ± 7.1	176.9–179.2	160.0–196.5
Body mass (kg)	77.8 ± 10.6	76.1–79.5	48.1–108.6
BMI (kg m^−2^)	24.56 ± 3.15	24.05–25.07	18.49–33.94
*Hematological and blood biochemical data*			
Hct (%)	46.1 ± 2.3	45.8–46.5	40.9–51.4
Hb (g dl^−1^)	15.6 ± 0.84	15.4–15.7	13.5–17.4
E (×10^12^ L^−1^)	5.13 ± 0.30	5.08–5.18	4.43–5.87
L (×10^9^ L^−1^)	5.98 ± 1.20	5.79–6.18	3.44–9.45
Na^+^ (mmol L^−1^)	140.1 ± 1.8	139.8–140.4	136.0–145.0
K^+^ (mmol L^−1^)	4.24 ± 0.29	4.20–4.30	3.57–5.06
Cr (μmol L^−1^)	89.0 ± 11.6	87.1–90.9	65.0–121.9
Alb (g L^−1^)	43.1 ± 3.1	42.6–43.6	36.9–52.3
*Hormonal data*			
T (nmol L^−1^)	20.8 ± 5.8	19.9–21.8	7.1–37.5
fT (nmol L^−1^)	0.328 ± 0.091	0.313–0.342	0.095–0.551
C (nmol L^−1^)	511 ± 130	489–532	161–852
fT/C ratio (×10^3^)	0.669 ± 0.257	0.626–0.711	0.217–1.495
SHBG (nmol L^−1^)	41.9 ± 19.1	38.8–44.9	9.2–93.1

Data are given as the mean ± SD, 95% confidence interval (95% CI), and minimum and maximum values (min–max).

BMI, body mass index; Hct, hematocrit value; Hb, hemoglobin concentration; E, erythrocyte count; L, leukocyte count; Na^+^, sodium concentration; K^+^, potassium concentration; Cr, creatinine concentration; Alb, albumin concentration; T, testosterone concentration; fT, free testosterone concentration; C, cortisol concentration; fT/C, free testosterone-to-cortisol ratio; SHBG, sex hormone-binding globulin concentration.

### Blood Collection

Overnight blood samples were taken from the antecubital vein at rest between 7:30 and 8:30 a.m. from all participants. Blood for serum CRP, AAG, FER, IL-6, total cholesterol (TC), triglycerides (TG), low- and high-density lipoproteins (LDL and HDL, respectively), total T, cortisol (C), and sex hormone-binding globulin (SHBG) concentrations was collected into plain tubes and left to clot for a minimum of 30 min at room temperature and then centrifuged at 4,000 rpm for 5 min. Blood for plasma IL-6 was collected in plain tubes containing EDTA and then centrifuged at 653 × *g* for 15 min at 4°C. Serum and plasma were stored at −80°C until analysis. With regard to CRP, AAG, and C, some data were unavailable and resulted in a reduced sample size for these variables (*n* = 142 for CRP and AAG and *n* = 145 for C and the fT/C ratio).

### Blood Analysis

The CRP and AAG concentrations were measured using the Siemens-Dade Behring BN ProSpec nephelometer (Marburg, Germany). Serum TC, TG, and LDL and HDL concentrations were determined with an enzymatic colorimetric method according to the manufacturer’s protocol using the Cobas c501 analyzer (Roche Diagnostics, Mannheim, Germany). Moreover, non-HDL concentration was calculated by subtracting the HDL cholesterol value from TC. Serum FER was measured with the chemiluminescence method using the Architect i1000SR analyzer (Abbott Laboratories, Chicago, IL, USA). Plasma IL-6 concentration was determined by enzyme-linked immunosorbent assay (ELISA) according to the manufacturer’s instruction (R&D Systems, Inc. Minneapolis, MN, USA). The detection limit for this measurement was 0.039 ng L^−1^ and the intra- and inter-assay coefficients of variation (CVs) were <8% and 10%, respectively.

All hormone measurements were performed in duplicate, and serum T, C, and SHBG were determined by an electrochemiluminescence immunoassay using the Cobas e411 analyzer (Roche Diagnostics, Mannheim, Germany) with detection limits of 0.09, 1.0, and 0.8 nmol L^−1^ for T, C, and SHBG, respectively. The intra- and inter-assay CVs for these assays were 3.4% and 5.9%, 1.2% and 1.6%, and 2.4% and 3.7% for T, C, and SHBG, respectively. The measurement method for T and C was standardized against the isotope dilution gas chromatography–mass spectrometry (ID GC/MS) reference method. SHBG measurement was standardized against the 1st International Standard for SHBG from the National Institute for Biological Standards and Control (NIBSC, code 95/560). Moreover, fT was calculated using the assumption-free empirical equations (25), as it was shown that this method is very useful in providing more detailed information about the androgen and anabolic/catabolic status of the body (26).

### Statistical Analysis

In this study, the bivariate correlations were evaluated using Spearman’s correlation coefficient because of the non-normal distribution of the inflammatory and lipid profile variables and the time spent on physical activity. Multiple regression analysis was performed to evaluate the independent contribution of androgens to inflammatory markers, adjusting for age, BMI, and blood lipids. We have calculated that a sample size of 134 individuals would be needed to obtain an effect size of *f* = 0.1 (conventionally attributed to a small effect) with five predictors, a significance level of 0.05, and power of 0.90. Non-normally distributed variables were log-transformed prior to analysis in order to reduce skewness of the data. The data points that deviated from the group means by more than 3 SDs were treated as outliers and excluded from further analysis. Then, the assumptions of the multiple regression analysis were verified and were not violated. The significance level was set at *p* ≤ 0.05, and all data are presented as the mean ± SD. The analyses were performed using STATISTICA software, version 10 (StatSoft, Inc., 2011; www.statsoft.com).

## Results

### Basic Characteristics

The means for the basic anthropometric, hematological, blood biochemical, and hormonal parameters of the studied group of subjects were all in the range of the reference values (**Table 1**), but there were individuals on the borderline of the normal ranges (hematocrit value, hemoglobin and albumin concentrations, erythrocyte and leukocyte counts, and all hormonal parameters). However, in these cases, further medical evaluation did not reveal any pathological conditions. Moreover, only one subject presented with T and fT concentrations slightly below the healthy adult male reference ranges, but even in this case, a late-onset hypogonadism (LOH) syndrome was not diagnosed because there were no symptoms suggestive of testosterone deficiency [see, e.g., (13)].

### Bivariate Correlations

**Figure 1:**
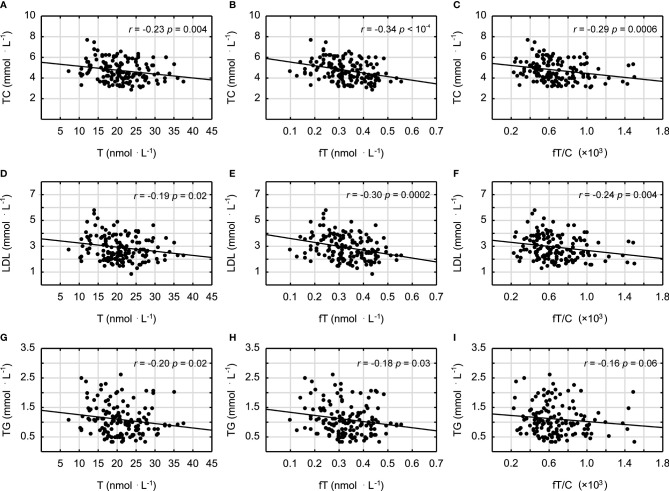
Spearman’s rank correlations between testosterone (T), free testosterone (fT), free testosterone-to-cortisol ratio (fT/C), and total cholesterol (TC) concentrations **(A–C)**; low-density lipoproteins (LDL) **(D–F)**; and triglycerides (TG) **(G–I)**.

**Figure 2:**
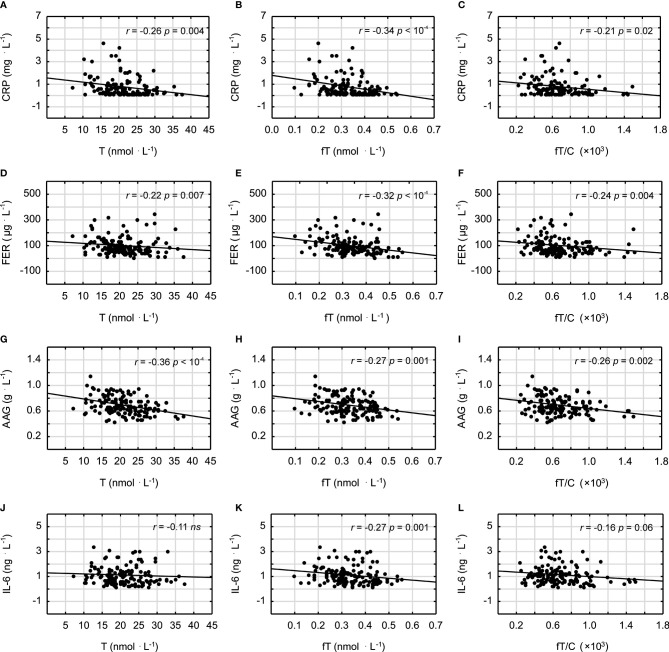
Spearman’s rank correlations between testosterone (T), free testosterone (fT), free testosterone-to-cortisol ratio (fT/C), and C-reactive protein (CRP) **(A–C)**; ferritin (FER) **(D–F)**; alpha-1-acid glycoprotein (AAG) **(G–I)**; and interleukin-6 (IL-6) **(J–L)**.

It was demonstrated that androgens (T and fT concentrations) and the fT/C ratio were significantly inversely correlated with the blood lipid profile in all studied men (in the case of the correlation between fT/C and TG, there was a clear tendency, *p* = 0.06) (**Figure 1**). Similarly the T and fT concentrations were all significantly inversely correlated with the inflammatory markers (**Figure 2**). The weakest relationship was observed between androgens and IL-6, however, the correlation between fT and IL-6 was significant, and there was also a tendency to significance between the fT/C ratio and the IL-6 concentrations (see **Figure 2**). On the other hand, there was no significant correlation between serum androgens and the HDL concentration (*p* > 0.05).

Simultaneously, age and BMI correlated positively with most of the inflammatory markers (CRP, AAG, FER, and IL-6) and the lipid profile variables (TC, LDL, non-HDL, and TG) and negatively with the androgen profile parameters (T, fT, and fT/C ratio) and HDL concentration. The only exceptions were a non-significant correlation between age and the AAG concentration and between age and the HDL concentration (see **Table 2**). Moreover, in the case of a correlation between age and the fT/C ratio, a clear tendency was observed (*p* = 0.07).

**Table 2:** Bivariate correlations between age and inflammatory markers, lipid profile, and androgen profile and between BMI and inflammatory markers, lipid profile, and androgen profile.

	Age (years)		BMI (kg m^−2^)	
	Spearman’s *r*	*p*-value	Spearman’s *r*	*p*-value
*Inflammatory markers*				
**CRP (mg L^−1^)**	0.39	<0.0001	0.49	<0.0001
**AAG (g L^−1^)**	0.11	ns	0.33	<0.0001
**FER (μg L^−1^)**	0.43	<0.0001	0.33	<0.0001
**IL-6 (ng L^−1^)**	0.53	<0.0001	0.36	<0.0001
*Lipid profile*				
**TC (mmol L^−1^)**	0.50	<0.0001	0.30	0.0002
**LDL (mmol L^−1^)**	0.49	<0.0001	0.33	<0.0001
**HDL (mmol L^−1^)**	0.06	ns	-0.20	0.02
**Non-HDL (mmol L^−1^)**	0.46	<0.0001	0.36	<0.0001
**TG (mmol L^−1^)**	0.30	0.0003	0.32	0.0001
*Androgen profile*				
**T (nmol L^−1^)**	−0.20	0.01	−0.31	0.0001
**fT (nmol L^−1^)**	−0.46	<0.0001	−0.34	<0.0001
**fT/C (×10^3^)**	−0.15	0.07	−0.18	0.03

BMI, body mass index; CRP, C-reactive protein; AAG, alpha-1-acid glycoprotein; FER, ferritin; IL-6, interleukin-6; TC, total cholesterol; LDL, low-density lipoprotein; HDL, high-density lipoprotein; non-HDL, non-high-density lipoprotein; TG, triglycerides; T, testosterone; fT, free testosterone; fT/C, free testosterone-to-cortisol ratio.

Moreover, we also determined the relationship between T, fT, and the fT/C ratio and physical activity level. It was demonstrated that there were significant positive correlations between all the androgen profile variables and the time spent on physical activity and sports (*r* = 0.23, *p* = 0.005; *r* = 0.25, *p* = 0.002; and *r* = 0.21, *p* = 0.01, respectively, for T, fT, and the fT/C ratio and time spent on physical activity).

### Multivariate Correlations

In order to assess the independent relationship between androgen status and inflammatory markers, we have created three linear regression models with CRP, AAG, FER, and IL-6 as separate dependent variables. As is shown in **Table 3**, the correlations between each inflammatory marker and the androgen profile parameters (T, fT, and fT/C ratio) were adjusted for age (model 1), age and lipid profile (model 2), and age, lipid profile, and BMI (model 3). In this multiple regression analysis, there were significant inverse correlations (or clear tendency for it) between all the androgen profile variables and the age- and lipid profile-adjusted CRP, AAG, and FER concentrations. However, when adjustment for BMI was made (model 3), these associations stayed significant only for the androgen profile variables and the AAG concentrations (see **Table 3**). The associations between fT and fT/C ratio and IL-6 concentration observed in the bivariate analysis (**Figure 2**) were no longer significant when controlling for other covariates (age, lipid profile, and BMI) (see **Table 3**).

**Table 3:** Associations between the inflammatory markers (CRP, AAG, FER, and IL-6) used as dependent variables and the androgen status (T, fT, and fT/C ratio), age, lipid profile (LDL, HDL, and TG), and BMI entered as independent variables in three linear regression models.

	T			fT			fT/C		
	*β*	*p*	a*R* ^2^	*β*	*p*	a*R* ^2^	*β*	*p*	a*R* ^2^
	**CRP**								
**Model 1:** age adjusted	−0.20	0.01	0.21	−0.22	0.01	0.20	−0.17	0.04	0.19
**Model 2:** adding LDL, HDL, and TG to model 1	−0.16	0.05	0.20	−0.17	0.06	0.19	−0.16	0.05	0.20
**Model 3:** adding BMI to model 2	−0.09	0.24	0.28	−0.11	0.17	0.28	−0.13	0.09	0.28
	**AAG**								
**Model 1:** age adjusted	−0.35	<0.0001	0.12	−0.28	0.002	0.06	−0.25	0.003	0.06
**Model 2:** adding LDL, HDL, and TG to model 1	−0.30	0.0003	0.18	−0.22	0.01	0.13	−0.21	0.01	0.14
**Model 3:** adding BMI to model 2	−0.25	0.003	0.21	−0.18	0.05	0.17	−0.18	0.03	0.18
	**FER**								
**Model 1:** age adjusted	−0.13	0.08	0.20	−0.18	0.03	0.21	−0.15	0.05	0.20
**Model 2:** adding LDL, HDL, and TG to model 1	−0.11	0.14	0.22	−0.17	0.05	0.23	−0.14	0.08	0.22
**Model 3:** adding BMI to model 2	−0.09	0.25	0.23	−0.15	0.08	0.23	−0.12	0.12	0.22
	**IL-6**								
**Model 1:** age adjusted	−0.002	0.98	0.29	0.005	0.94	0.29	−0.06	0.43	0.29
**Model 2:** adding LDL, HDL, and TG to model 1	−0.02	0.80	0.28	0.006	0.94	0.28	−0.08	0.28	0.28
**Model 3:** adding BMI to model 2	0.02	0.80	0.29	0.04	0.65	0.29	−0.06	0.42	0.29

BMI, body mass index; T, testosterone; fT, free testosterone; fT/C, free testosterone-to-cortisol ratio; CRP, C-reactive protein; AAG, alpha-1-acid glycoprotein; FER, ferritin; IL-6, interleukin-6; LDL, low-density lipoprotein; HDL, high-density lipoprotein; TG, triglycerides; β, standardized beta coefficient; p, significance value; aR^2^, adjusted coefficient of determination.

## Discussion

The findings presented in this study demonstrated that a lower serum T concentration is related to chronic low-grade inflammation and an unfavorable blood lipid profile, which may have important impacts on increasing atherosclerosis risk in humans. This statement is supported by the negative correlations between the markers of androgen status (T, fT, and fT/C ratio) and blood lipid profile (TC, LDL, and TG) and between the markers of androgen status and the inflammatory markers (CRP, FER, AAG, and IL-6) (see **Figures 1** and **2**). However, it should be pointed out that, although the associations between androgens and the acute phase proteins (CRP, AAG, and FER) were independent of age and blood lipid profile (**Table 3**), only the correlations between the markers of androgen status and AAG were independent of BMI. It must also be acknowledged that these results, which are based on correlations, do not infer causation.

### Androgens and Adipose Tissue

The development of chronic low-grade inflammation depends on both visceral fat content and sex hormones [see, e.g., (27)], and the interactions between fat mass and androgens may have important outcomes on its progression (28). In the present study, we have demonstrated that the relationship between T and the inflammatory markers (CRP and FER) is not independent of BMI, which suggests that this association is conditioned by body fat (**Table 3**). The unfavorable impact of a higher body mass on the inflammatory status is well known (29), and the chronic low-grade inflammatory state may exist even when the fat content is within the physiologically acceptable limits (11). Nevertheless, from the results of the present study, it could be postulated that a higher body fat adversely influences not only the inflammatory status (by increasing CRP, AAG, FER, and IL-6), which was observed in our earlier report (11), but also the androgen status (decreasing T, fT, and fT/C ratio). The significant bivariate correlations between BMI and the markers of both androgen and inflammatory profiles (see **Table 2**) support such conclusions. They also correspond to the results of Svartberg et al. (30), who reported that the inverse correlation between T and cIMT (carotid intima–media thickness, a marker of artery atherosclerosis) was BMI-dependent.

The negative relationship between T concentration and fat mass has often been demonstrated (31, 32). Moreover, it was stated that a change in BMI from “non-obese to obese” may be equivalent to a 15-year fall in the T concentration (32) and that interventions reducing BMI are expected to increase serum T in men (33). Although the exact mechanism of the negative effect of a higher fat mass on the T concentration is not fully understood, it seems that hypothalamic–pituitary inhibition of gonadotropin release takes place through different central and peripheral signals. However, it should also be pointed out that there is a mutual relation between fat mass and androgens since androgens have been shown to affect a number of adipose tissue functions including adipocyte differentiation, lipid metabolism, and their secretory activity (34). Additionally, testosterone replacement therapies have been proven to be effective in decreasing adipose tissue mass (35), and this effect may be related to lipoprotein lipase activity inhibition, decreased triglyceride accumulation, and lipolysis stimulation [see, e.g., (36)]. These data are in accordance with the inverse relationship between T and the lipid profile variables (TC, LDL, and TG) observed in this study (**Figure 1**). On the other hand, the results showing that testosterone promotes the commitment of the mesenchymal pluripotent cells to the myogenic lineage and inhibits their differentiation into the adipogenic lineage (37) were used to elucidate the reciprocal effects of androgens on the muscle and fat mass in men, and they may also be considered as the potential explanation of the inverse correlation between androgens and BMI presented in this study (**Table 2**) and by others (31).

### Androgens and Inflammation

Adipose tissue may be involved in enhanced oxidative stress and inflammation with aging (11), but the results of this study suggest that the inflammatory process may be independently associated not only with body fat and age but also with androgen level. We based this conclusion on the observation of the significant correlation between the markers of androgen profile and the AAG concentration in the multiple regression analysis including age and BMI as relevant and independent potential confounders (see **Table 3**). Although the physiological mechanisms behind this relationship are unclear, the direct influence of androgens on the different stages of the inflammatory process has been reported (7, 8), and Norata et al. (38) have demonstrated that, in endothelial cells, the anti-inflammatory role of androgens is exerted through the inhibition of the nuclear factor kappa B (NF-κB)-dependent expressions of adhesion molecules, cytokines, and proteases. This effect may occur through a testosterone-induced decrease of the expression of Toll-like receptor 4 (TLR4), which is known to stimulate different signaling pathways such as the NF-κB pathway (39). Suppression of NF-κB in endothelial cells by testosterone was later confirmed by Jin et al. (40), who also demonstrated that the physiological concentration of T alleviates the downregulation of TNF-α-mediated tissue factor pathway inhibition (TFPI), resulting in a reduction of blood coagulation.

Although AAG was first described in 1950 [see (41)], its complex regulation and immunomodulatory effects are still under extensive investigation (42). Taking into account that we have reported a significant positive correlation between the AAG concentration and BMI [see **Table 2** and “Results” in (11)], one may suggest that a higher body fat stimulates AAG production by hepatocytes through the secretion of pro-inflammatory cytokines, mainly TNF-α and IL-6. Nevertheless, the multiple regression analysis revealed that the best predictive parameter for AAG was testosterone (*β* = −0.25, compared to *β* = 0.20 and *β* = −0.17 for BMI and age, respectively), suggesting a connection between these two parameters that is unrelated to adipose tissue–testosterone interactions. The exact mechanism by which androgens may influence the AAG level is unclear, but based on the above-mentioned effects of androgens on the inflammatory process, it may be suggested that testosterone may diminish AAG production through its inhibitory action on inflammatory cytokine synthesis. The negative association between T concentration and the inflammatory markers, which is frequently reported (12, 43), supports this conclusion. On the other hand, the BMI-independent relationship between androgens and AAG could be explained by the fact that extrahepatic AAG expression occurs in cell types other than the adipose tissue and may be regulated by inflammatory mediators, as in hepatocytes (41).

It should also be mentioned that AAG was demonstrated to maintain metabolic homeostasis and to suppress inflammatory processes (44), similar to the functions of IL-6 and CRP that may also be elevated in the absence of an inflammatory state and exert anti-inflammatory effects, as recently reviewed by Del Giudice and Gangestad (45). However, in our subjects, the higher concentrations of AAG, CRP, and IL-6 were positively correlated with a higher FER, an oxidative stress marker (*r* = 0.20, *p* = 0.02; *r* = 0.33, *p* < 10^−4^; and *r* = 0.17, *p* = 0.05, respectively), a higher BMI, and a lower androgen profile (see *Results*), which, together, represent a hallmark of the inflammatory state, especially in the absence of other medical conditions. It seems that the role of AAG in inflammatory progression needs to be further investigated, also in view of the recent data that demonstrated the importance of IL-6 and IL-6R (interleukin-6 receptor) in cardiovascular diseases (46, 47).

### Androgens and Aging

It is widely accepted that aging per se decreases the T and fT concentrations in men (48), especially after the fifth decade of life (49), but the degree of this process also depends on the health status of the studied men (50). In the European Male Ageing Study, it was demonstrated that, in older men, with over 4 years of follow-up, T decreases annually by 0.1 nmol L^−1^ and fT by 3.83 pmol L^−1^ (0.04% and 0.77%, respectively) (31). However, Sartorius et al. (51) reported that, in aged men characterized by “very good or excellent” health, serum sex steroids are not reduced. This finding led the authors to the conclusion that the changes in the androgen levels observed in other studies may be rather attributable to comorbidities in the aging population than to the aging process itself. This conclusion seems to be in contrast to the results presented in this paper. Although we have demonstrated that physically active men tend to have higher sex hormone concentrations than do the inactive ones (see *Results*, *Bivariate Correlations*), there was a significant negative correlation between age and the androgen status, especially the fT concentration, i.e., the biologically active form of gonadal androgens (see **Table 2**). These findings suggest, according to the classic view (49), that the T and fT concentrations diminish with aging, however, at the same time, it may be postulated that lifelong moderate-intensity exercise training may attenuate this process.

### Androgens and Exercise

The data regarding the effect of exercise training on gonadal androgens in aging males are, however, inconsistent (52, 53). Nevertheless, based on our earlier studies (20, 26, 54, 55), we can suggest that the basal T and fT concentrations change in response to exercise training accordingly to the applied training load. Therefore, it can be postulated that a low-to-moderate training load leads to an increase in the T concentration (20, 54), a moderate-to-heavy training load does not change it (26), and a heavy-to-maximal training load leads to a decrease in its concentration (55). This concept was recently supported in the review by Matos et al. (56), and it can also explain why Lovell et al. (52) did not observe any changes in the resting T and fT concentrations during training of progressively increasing loads in older men, whereas Hayes et al. (53) found enhanced basal T and fT concentrations after training of shorter durations and lower total loads (although partly of higher intensities).

The training-induced changes in the androgen concentrations, regardless of whether they are related to the direct stimulation of the HPG axis or to the effects of body fat–androgens interactions, may be of great importance because we have demonstrated that they are inversely correlated with markers of inflammation and blood lipids (**Figures 1** and **2**). It should also be emphasized that a lower T level is associated with muscle weakness (57) and a faster VO_2_ peak decline during aging (58), which decreases the muscle power-generating capabilities and the willingness to engage in spontaneous physical activity. The reduced physical activity might, in turn, further compromise systemic T availability and contribute to the unfavorable changes in the inflammation and blood lipid profile. This hypothesis, together with our results, is consistent with previous studies showing that T is negatively correlated with the CRP (59), TC (60), and TG concentrations (60, 61) and positively with the HDL concentration (61) and that men with lower T concentrations have unfavorable lipid profile (HDL < 0.90 and TG > 1.80) (62). Although the correlation between T and HDL was not significant in this paper (*p* > 0.05), it should be noted that exercise training may induce an enhanced HDL concentration. One may speculate that a moderately higher level of physical activity affects positively both the testosterone and HDL concentrations, however, recent data have indicated that a more important risk factor for cardiovascular events is HDL cholesterol efflux capacity (63), which could be influenced by gonadal androgens (64).

Based on the above literature data, it may be inferred that the correlation between androgens and inflammatory markers observed in this study is not accidental. Simultaneously, we are aware of the limitations of our study, especially that related to the cross-sectional design of the research and the limited number of studied men, which impeded us from establishing a firm causality between the androgen status and the inflammatory markers and blood lipid profile. We also acknowledge that measurements of the apolipoproteins and oxLDL (oxidized LDL) and determination of anti-inflammatory markers would reinforce our results. Nevertheless, the multiple regression analysis, including androgen, inflammatory, and blood lipid profile, in this moderately large group of men (see **Table 3**) enabled us to claim that testosterone may affect the inflammatory process independently of adipose tissue and age, which confirms other findings (43) and suggests a direct connection between the T and fT concentrations and inflammatory markers.

## Conclusion

Summing up, we have shown that chronic low-grade inflammation might be linked to lowered androgen levels in men. Based on the results of this study and on previously published data, we have concluded that a lowered androgen profile is related to a reduced inhibition of inflammatory cytokine synthesis, which leads to an enhanced production of acute phase proteins. Accordingly, we postulate that a low serum T concentration should be considered as an independent risk factor in the development of atherosclerosis and cardiovascular diseases. Moreover, the positive correlation between testosterone and physical activity level suggests that exercise training may reduce the age-related decrease in gonadal androgens, which seems to be one of the main beneficial effects (anti-inflammatory one) of physical activity in aging men.

## Data Availability Statement

The raw data supporting the conclusions of this article will be made available by the authors, without undue reservation.

## Ethics Statement

The studies involving human participants were reviewed and approved by the Local Ethical Committee at the Regional Medical Chamber in Krakow, Poland (opinion no. 48/KBL/OIL/2009). The participants provided written informed consent to participate in this study.

## Author Contributions

MG and JAZ contributed to the conception and design of the study. MG, JM, JZ-B, KD, JKK, and JAZ performed the experimental procedures and data analysis. MG performed the statistical analysis and wrote the first draft of the manuscript. MG, JM, JKK, and JAZ wrote sections of the manuscript. All authors contributed to the article and approved the submitted version.

## Funding

This work was supported by the European Union from the resources of the European Regional Development Fund under the Innovative Economy Programme (grant no. POIG.01.01.02-00-069/09) and by funds from the University School of Physical Education in Krakow, Poland, for the statutory research in 2015–2018 awarded to JAZ (grant no. 120/BS/KFiB/2017) and to MG (grant nos. 75/BS/KFiB/2015 and 121/BS/KFiB/2017).

## Conflict of Interest

The authors declare that the research was conducted in the absence of any commercial or financial relationships that could be construed as a potential conflict of interest.

## Publisher’s Note

All claims expressed in this article are solely those of the authors and do not necessarily represent those of their affiliated organizations, or those of the publisher, the editors and the reviewers. Any product that may be evaluated in this article, or claim that may be made by its manufacturer, is not guaranteed or endorsed by the publisher.
